# Thanksgiving to Yeast, the HMGB Proteins History from Yeast to Cancer

**DOI:** 10.3390/microorganisms11040993

**Published:** 2023-04-11

**Authors:** Mónica Lamas-Maceiras, Ángel Vizoso-Vázquez, Aida Barreiro-Alonso, María Cámara-Quílez, María Esperanza Cerdán

**Affiliations:** 1 Centro Interdisciplinar de Química y Biología (CICA), As Carballeiras, s/n, Campus de Elviña, Universidade da Coruña, 15071 A Coruña, Spain; 2 Instituto de Investigación Biomédica de A Coruña (INIBIC), As Xubias de Arriba 84, 15006 A Coruña, Spain; 3 Facultad de Ciencias, A Fraga, s/n, Campus de A Zapateira, Universidade da Coruña, 15071 A Coruña, Spain

**Keywords:** yeast, molecular methods, research hits, HMGB proteins, cancer, interactome

## Abstract

Yeasts have been a part of human life since ancient times in the fermentation of many natural products used for food. In addition, in the 20th century, they became powerful tools to elucidate the functions of eukaryotic cells as soon as the techniques of molecular biology developed. Our molecular understandings of metabolism, cellular transport, DNA repair, gene expression and regulation, and the cell division cycle have all been obtained through biochemistry and genetic analysis using different yeasts. In this review, we summarize the role that yeasts have had in biological discoveries, the use of yeasts as biological tools, as well as past and on-going research projects on HMGB proteins along the way from yeast to cancer.

## 1. Introduction

Biological discoveries affecting human life have been made using model systems that are easy to grow and manipulate in laboratories. The bacteria *Escherichia coli*, *Saccharomyces cerevisiae*, and *Schizosaccharomyces pombe* yeasts; the nematode *Caenorhabditis elegans*; the zebrafish *Danio rerio*; the fly *Drosophila melanogaster*; and the mouse *Mus musculus* are widely known as studied models [1]. Without any doubt, unicellular organisms—prokaryotes and eukaryotes—were the pioneers among them.

During the 20th century, yeasts were widely used in research. Initially, they were used in genetic approaches based on the study of the phenotypes of their mutants; then, yeast mutants were used to clone genes of other species by complementation. Now, yeasts are widely known as cellular factories to express heterologous proteins [2,3]. Knowledge of the molecular biology of yeasts and the development of recombinant DNA and yeast transformation techniques also allowed for their use as molecular tools and in medical research [4].

In the era of omics, we cannot forget that the *S. cerevisiae* eukaryotic genome was the first to be fully sequenced; a few years later, a set of yeast strains with deletions of most of its annotated open reading frames (ORF) was made available and this knowledge opened new doors to numerous functional analyses of genes that were completely unknown until then [5,6,7]. 

Despite the time that has elapsed from their first uses, nowadays yeasts are still powerful allies in biological research; as an example of their current applications, we summarize the history of the study of HMGB proteins in our laboratory, which over the years has led us from the study of the transcriptional regulation of hypoxic genes to the search for molecular markers and therapies for cancer.

## 2. Conventional and “Non-Conventional” Yeast Models

**Figure 1:**
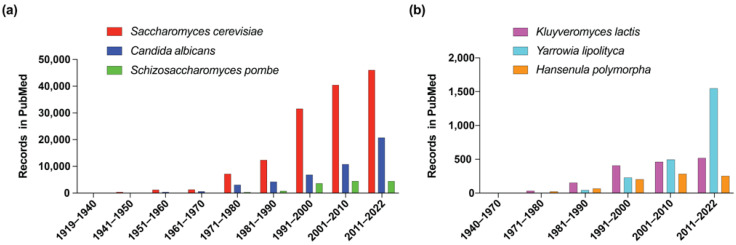
Evolution of yeast publications in PUBMed. (**a**) Conventional and (**b**) less-conventional yeasts.

Among the yeasts most used as biological models, we must mention *S. cerevisiae*, which divides by budding; *S. pombe*, which divides by medial fission, and is thus commonly referred to as “the fission yeast”; and *Candida albicans*, which causes millions of mucosal and systemic infections per year. The first paper that can be found in PUBMed related to yeasts was published in 1919; in the paper, authored by Leslie Herbert Lampitt from the University of Birmingham, *S. cerevisiae* is used to study nitrogen metabolism [8]. Since then, the number of publications in PUBMed referencing these three yeasts increased substantially (Figure 1). In addition, other non-conventional yeasts, which have many applications in biotechnology—such as *Kluyveromyces lactis*, *Yarrowia lipolytica*, and *Hansenula polymorpha*—have also been extensively studied, although the number of published papers is minor by order of magnitude (Figure 1). The use of non-conventional yeasts was early envisaged as a fruitful alternative for heterologous protein production, since some species exhibit favorable traits such as high-level secretion or strong and tightly regulated promoters, offering significant advantages over *S. cerevisiae* [9]. Nowadays, specific yeast strains are considered to hold potential value for effective metabolic engineering in the new era of synthetic biology to generate effective yeast cell factories [10].

Proliferative cells from tumors preferentially metabolize glucose to lactate, even in the presence of oxygen—a process known as the Warburg effect. In this process, the switch between different isoforms of the pyruvate kinase enzyme, which catalyzes a rate limiting step of glycolysis, is determinant [11]. These tumor cells have an energetic metabolism closer to a yeast model such as *S. cerevisiae*, in which fermentative metabolism predominates over respiration due to catabolite repression, which diminishes the expression of genes related to cellular respiration [12]. However, slow-cycling tumor cells have a metabolism more dependent on mitochondria and oxidative phosphorylation [13,14], and increasing evidence demonstrates that cancer stem cells rely preferentially on oxidative phosphorylation for obtaining energy [15]. In *K. lactis*, catabolite repression is absent and the metabolism is predominantly respiratory [16,17], similarly to slow-cycling tumor cells, cancer stem cells, or non-cancerous cells.

## 3. Yeasts as Biological Tools

Traditionally, yeasts have been used as a “factory” to produce molecules of therapeutic value, such as vaccines or products of industrial interest. This approach has been facilitated by the ease of their cultivation and handling, the possibility of genetic modifications to produce heterologous proteins, and because many of the selected yeast strains are safe and belong to the category of harmless organisms known as GRAS (generally recognized as safe), a concept created in 1958 by the American FDA (Food and Drug Administration) [18]. However, beyond their biotechnological use, yeasts have allowed the development of diverse screening methods quite common in molecular laboratories, including the yeast two-hybrid system or the yeast surface display.

The yeast two-hybrid (Y2H) system was first published in 1989 [19]. Since then, it has become a powerful and affordable tool for the detection of protein–protein interactions in the postgenomic era. The detection of a given protein–protein interaction is possible through the co-expression in a yeast strain, which carries the necessary mutations to make selections, of two chimeric proteins. If there is interaction, they reconstruct a transcriptional activator with its DNA-binding domain directed to a promoter, and its activation domain is able to activate the transcriptional machinery so that this positive interaction can be recognized, and even quantified, by the expression of a reporter gene [20]. The two-hybrid method has been the starting point of many other variants. One is the yeast mono-hybrid that allows the detection of interactions between protein and DNA [21]. The triple hybrid (Y3H) technology was originally developed for studying protein–small molecule interactions [22]. The Y3H is an extension of the Y2H but introducing a third hybrid component, usually a small molecule that can make possible or interfere with the protein–protein interaction, or a RNA molecule allowing the detection of protein–RNA interactions. All these methods allow the study of interactions between proteins of any other biological origin in the yeast model; for this reason, the Y2H has been extensively used in pharmacological screenings for novel drugs [23,24].

Engineered yeasts with functional proteins displayed on the surface have many potential applications, not only for high-throughput library screening but also in biocatalysis, as biological sorbents, oral vaccines, etc. [25]. Interestingly, proteins anchored in the membrane are more resistant to degradation or denaturation by extreme pH or elevated temperature; therefore, they maintain functional properties better than the corresponding free forms. For biocatalysis, an additional advantage of cell-surface display technology is that it can be used with substrates that cannot enter the cell, for instance, large polymers of cellulose or hemicellulose. For advanced biocatalysis, the multi-enzyme cell surface co-display also allows the expression at a short distance, compatible with the efficient transfer of substrates, of the whole set of enzymes involved in a metabolic pathway [26]. It is important to highlight that when used in high-throughput library screening, this technology allows easy recovery of the proteins or small molecules bound to the target surface protein by dissociation and filtration or centrifugation, avoiding other necessary high-cost processes of purification when molecules are inside the cells.

Although yeast cell surface display was first developed in *S. cerevisiae* [27], it was later adapted to other yeasts, such as *Yarrowia lipolytica* [28] or *Pichia pastoris* [29,30]. In surface display, the protein or peptide of interest is expressed in yeast fused to a secretory signal and to an anchor protein, which will guide it along the secretory pathway and immobilize it in the cell wall, respectively. Several anchors and improvements can be used in yeast surface cell display, as recently reviewed [31,32].

The use of yeast systems as biological tools is of great relevance in the study of molecular mechanisms of cancer-related processes, the testing of new anti-cancer medicaments, and the characterization of resistance mechanisms [33,34,35,36].

## 4. Outstanding Milestones for Yeast Thanksgiving and Their Relation to Cancer Research

It is impossible to make a detailed summary of all the scientific discoveries in which yeasts have been involved, and of the many scientists and laboratories that participated in these studies. The understanding of many of these processes has later been transferred to multicellular organisms, making it possible to explain complex mechanisms that allow cell homeostasis and that, when deregulated, cause a wide variety of diseases.

There is a big spectrum of more than 200 different human cancers but, theoretically, all the mechanisms that allow a normal cell to transform into a cancerous one by dividing and changing its microenvironment to generate the tumor have common characteristics. This is because cancer cells accumulate defects in regulatory circuits, which govern normal cell proliferation and homeostasis. Hanahan and Weinberg enumerated all these shared traits and originally defined six cancer hallmarks [37] that were later extended to eight and two enabling capabilities. With the advances of research in various fields, a new core of cancer hallmarks has recently been proposed: sustaining proliferative signaling, evading growth suppressors, non-mutational epigenetic reprogramming, avoiding immune destruction, enabling replicative immortality, tumor promoting inflammation, polymorphic microbiomes, activating invasion and metastasis, inducing or accessing vasculature, senescent cells, genome instability and mutation, resisting cell death, deregulating cellular metabolism, and unlocking phenotypic plasticity [38].

**Figure 2:**
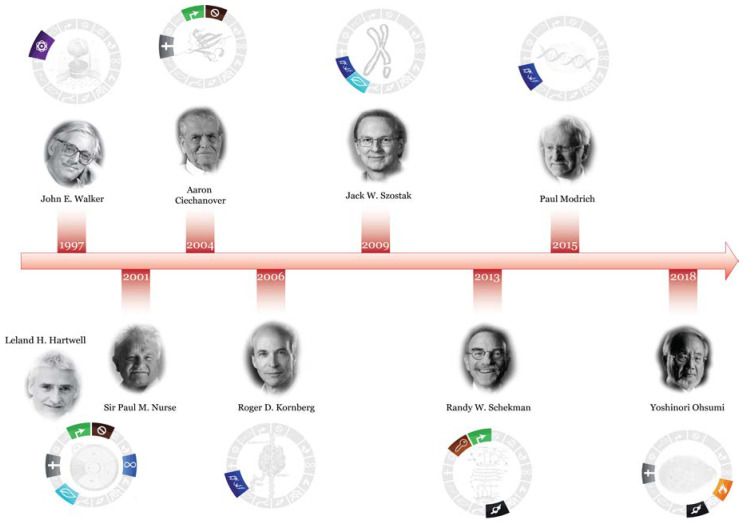
Yeast research in Nobel Prizes and their relation to cancer hallmarks. Symbols for cancer hallmarks are from [38].

Despite their simplicity compared with mammalian cells, the discoveries and applications of research carried out using yeast cells have had a profound impact on the study of cancer (Figure 2), as shown with some examples below, and do not represent an exhaustive repertoire of all the implications of yeast research in this field.

Complex structures that the cell needs to obtain energy for its metabolic reactions or that are necessary for the synthesis of proteins were first solved in bacteria or yeast. The structure of the ATPase from *S. cerevisiae* mitochondria, solved in 1997 after John E. Walker received the Nobel Prize in Chemistry, validated the earlier proposal of a rotational motion of the F1 domain of this protein, which behaves as a molecular motor for energy conversion of proton gradient to ATP [39]. Cancerous cells depend on ATP production by glycolysis or oxidative phosphorylation to survive. In multiple cancers, slow-dividing cancer cells generate ATP via mitochondrial oxidative phosphorylation [40]. Besides, cancer stem cells, which are resistant to regular chemo- and radiotherapy, also rely on oxidative phosphorylation for energy supply [40]. This knowledge can be used in therapy; i.e., Gboxin is an oxidative phosphorylation inhibitor that acts on Complex V (ATP synthase) and targets glioblastomas [14]. 

Venkatraman Ramakrishnan, Thomas A. Steitz, and Ada E. Yonath, for the Nobel Prize in Chemistry 2009, studied the structure and function of the prokaryotic ribosome; only two years later, the crystal structure of the eukaryotic *S. cerevisiae* ribosome was published by Adam Ben-Shem [41]. A close interconnection between ribosome biogenesis and cell proliferation has been reported, showing that up-regulated ribosome production down-regulates p53 expression and activity, thus facilitating neoplastic transformation [42]. Research on eukaryotic ribosome biogenesis and assembly was facilitated by the development of assays for pre-rRNA processing and by genetic screens for ribosome assembly factors in yeast, as well as by methods to purify and characterize assembly intermediates [43]. 

The discovery of the molecular basis of eukaryotic transcription is highly associated with yeast. In 2006, the Nobel Prize in Chemistry was awarded to Roger D. Kornberg for his research in this field [44,45,46,47]. Transcription controls diverse aspects of genomic integrity by different mechanisms, which might be responsible for transcription-associated mutation (TAM) and transcription-associated recombination (TAR) [48]. It was reported that elevated levels of transcription in yeast are associated with increased spontaneous mutation rates [49]. Evidence for TAR in eukaryotes was first shown with the *HOT1* gene of *S. cerevisiae* [50]. Increasing evidence also supports that oncogenes, such as RAS, and targeted cancer treatments, such as bromodomain and extra-terminal motif (BET) bromodomain inhibitors, increase global transcription, leading to R-loop accumulation, transcription–replication conflicts, and the activation of replication stress responses [51].

Yeast has also been a good model for studies on DNA repair mechanisms [52,53,54,55]. In 2015, the Nobel Prize in Chemistry was awarded jointly to Tomas Lindahl, Paul Modrich, and Aziz Sancar for mechanistic studies of DNA repair [56]. Cancer cells tend to harbor increased mutations in DNA Damage Response (DRR) genes, which restore the damaged DNA, and often have fewer DDR pathways than normal cells; thus, they become more susceptible to compounds inhibiting those pathways compared to normal cells, a characteristic that is useful in cancer therapy [57].

The use of yeast mutants has allowed the characterization of important biological processes. Over his career, Paul Nurse used *S. cerevisiae* and. *S. pombe* models in many experiments to study cell cycle control [58,59]; finally, in 1987, he cloned the cdc2 human homolog by complementation of the *S. pombe* mutant [60]. Meanwhile, Lee Hartwell was using *S. cerevisiae* defective mutants in the checkpoint, controlling the rate of progression through S phase in response to DNA damage [61]. Therefore, the Nobel Prize in Physiology or Medicine they shared in 2001 was definitively a “yeast prize” [62]. Undoubtedly, the discovery of genes that control cell division in yeast and other eukaryotes had clear implications in cancer research [63]. 

Aaron Ciechanover used yeast mutants to clone human homolog genes involved in protein ubiquitination, previously studied in the budding yeast [64], and obtained the Nobel prize in Chemistry in 2004 together with Avram Hershko and Irwin Rose. The ubiquitin–proteasome system degrades abnormal or redundant proteins and regulates cell proliferation, differentiation, metabolism, autophagy, and other physiological or pathological processes including cancer [65]. Key substrates of the cell cycle are regulated by ubiquitination mediated by the APC protein complex, and Cdc20 and Cdc20 homolog 1 (Cdh1) are coactivators responsible for ligating substrates and activating the ubiquitin ligase activity of APC, forming two different E3 ubiquitin ligase complexes, APCCdc20 and APCCdh1 [66]. Cdc20 is overexpressed in various cancer stem cells and malignant tumors, and its inhibition has been proposed as a targeted therapy for cancer patients [67].

The discovery of the role of telomeres in maintaining chromosome integrity and genetic stability, as well as their implications for cellular senescence, was also facilitated using yeast models. Jack W. Szostak carried out pioneering studies in yeast that led him to share the Nobel Prize in Physiology or Medicine with Elizabeth H. Blackburn and Carol W. Greider in 2009 [68]. Genomic instability is the main cause of many of the alterations that give rise to cancer hallmarks, and the length and stability of telomeres is a crucial factor also widely studied in yeast. Elimination of telomeric DNA in *S. cerevisiae* caused those cells to undergo an arrest of cell cycle progression due to activation of the DNA damage checkpoint [69]. The genes (EST) encoding different telomerase subunits, as well as the template RNA component, were first characterized in *S. cerevisiae* [70,71,72]. Replicative senescence elicited by activation of the checkpoint response is a state of stable, terminal cell-cycle arrest that acts as a barrier against tumorigenesis. However, downregulation of the checkpoint increases genomic instability, which, if coupled with re-stabilization of telomeres, can drive tumorigenesis [73]. The checkpoint response can be overcome either through mutational inactivation of its components or through “adaptation”, which is a phenomenon originally described in yeast [69]. In cancer cells, re-stabilization of telomeres is frequently caused by reactivation of telomerase, although it can also be produced by recombination-based mechanisms, called “alternative lengthening of telomeres” (ALT), which were also first described in yeast [74].

In the 1970s, Randy Schekman initiated a study on the genetic basis of vesicle traffic using yeast as a model system; he identified yeast mutants with defective transport machinery that caused phenotypes characterized by vesicles to pile up in certain parts of the cell. He also characterized the mutated genes, clustering them in three classes that control different facets of the cell’s transport system [75]. The Nobel Prize in Physiology or Medicine 2013 was awarded jointly to James E. Rothman, Randy W. Schekman, and Thomas C. Südhof for their discoveries in the field of vesicle traffic, a major transport system in our cells [76]. Carcinogenesis from cells organized in epithelia involves the loss of cell polarity, alteration of polarized protein presentation, dynamic cell morphology changes, increased proliferation, and increased cell motility and invasion. Although mutations in vesicle trafficking proteins may not be direct drivers of malignant transformation, the regulators of membrane vesicle trafficking are essential mediators of changes that drive cancer cell biology [77].

In 2016, the Nobel Prize in Physiology or Medicine was awarded to Yoshinori Ohsumi for his discoveries of the mechanisms of autophagy, in which baker´s yeast was used [78]. Autophagy is a physiological cellular process for the degradation of damaged proteins and organelles that has important function during development, cell death, and tumor suppression [79]. In cancer biology, autophagy plays dual roles in tumor promotion and suppression [80]. Tumor suppressor factors are negatively regulated by mTOR and AMPK, resulting in the induction of autophagy and suppression of cancer initiation [81]. In contrast, oncogenes may be activated by mTOR, class I PI3K, and AKT, resulting in the suppression of autophagy and enhancement of cancer formation [82]. The cancer microenvironment—including hypoxia, inflammation, and cytokines—is also affected by autophagy, which supplies the demand for cellular energy and prevents cytotoxicity (reviewed in [80]). In early metastasis, autophagy also reduces invasion and migration of cancer cells from origin sites. However, in advanced stages of metastasis, autophagy acts in a pro-metastatic role via promotion of cancer-cell survival and colonization in secondary sites (reviewed in [80]).

In relation to cancer hallmarks and by its connection to HMGB proteins, it is also interesting to mention the importance of yeast studies in the elucidation of the rapamycin signaling pathway (mTOR) [83,84,85]. mTOR is conserved from yeast to human and senses coordinately diverse signals such as nutrients, oxygen, hormones, and stress, being deregulated in multiple age-related diseases including cancer. mTOR regulates proliferation and lifespan by controlling gene expression, ribosome biogenesis, proteostasis, and mitochondrial metabolism; therefore, deregulation of mTOR pathways also causes deregulation of cellular metabolism [86]. Besides, rapamycin is a potent immunosuppressant that blocks the G1/S transition in antigen-activated T cells and in yeast [87], which connects mTOR activity and immune-scape. mTOR also controls autophagy [85], which is enhanced in cancer [88], and has been considered a counterbalance to programed cell death, which allows cancerous cells to resist cell death [89].

## 5. The HMGB Proteins in Cancer

**Figure 3:**
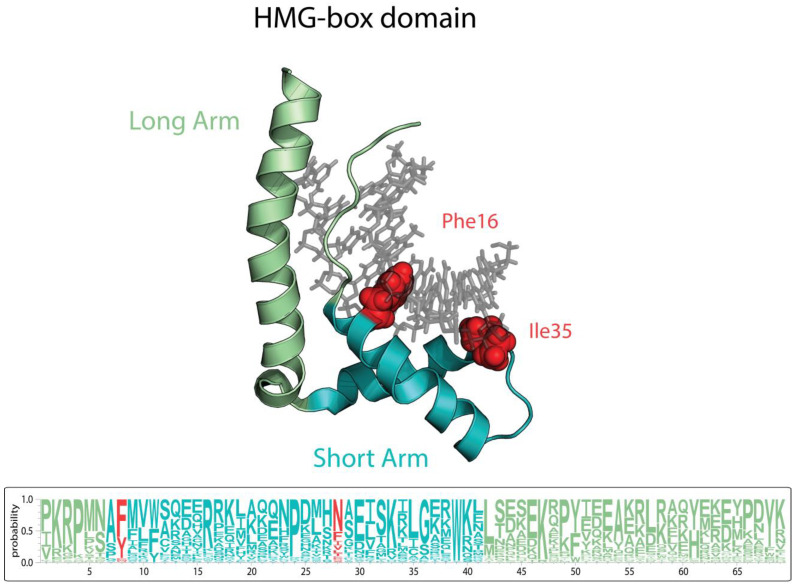
Structure of the HMG-box domain based on Hmgb1 protein structure PDBID: 2gzk and alignments of HMGB proteins. The amino acid frequency for each position of the HMG-box domain is represented in Logo format based on the multiple alignment available in Supplementary File S1. Amino acid sidechains that intercalate between the DNA base steps to induce the DNA kinks are indicated in red.

High Mobility Group B (HMGB) proteins are characterized by the presence of one or more HMG-box domains of 65–85 amino acids. The HMG-box domain has a characteristic L-shaped fold formed by three α-helices with an angle of ≈80° between the two arms (Figure 3). HMGB proteins are conserved over their evolution from unicellular to multicellular organisms (reviewed in [90]) and carry out diverse nuclear, cytoplasmic, and extracellular functions. There are four HMGB human proteins, with HMGB1 and HMGB2 being the most studied. Although they have similar amino acid sequences, their functions do not overlap [91].

HMGB1 cellular localization depends on post-translational modifications [92]. Acetylation/deacetylation of the nuclear localization signals of HMGB1 causes a shuttle between the nucleus and the cytoplasm; other modifications, such as methylation, N-glycosylation, phosphorylation, and oxidation, can regulate the translocation and release of HMGB1 to the extracellular space in response to various stresses (recently reviewed in [93]). HMGB1 has three different redox forms (all-thiol-HMGB1, disulfide-HMGB1, and oxidized HMGB1) in reference to the reduced or oxidized state of three conserved cysteine’s: Cys23 and Cys45, which can form intermolecular disulfide bonds, and Cys106 [94,95,96].

In the nucleus, HMGB proteins bind DNA through their HMG-boxes and regulate multiple genomic processes such as DNA damage repair, nucleosome sliding, telomere homeostasis, and transcription; recent evidences demonstrate that they also bind RNAs. Therefore, nuclear functions of HMGB proteins have broad regulatory impact on cells in normal and disease states (reviewed in [97]). HMGB1 regulates autophagy and apoptosis [97]. In the cytoplasm, disulfide-HMGB1 binds to Beclin 1 and affects autophagosome formation [98]. HMGB1 also participates in mitochondrial quality control [99] and in mitochondrial DNA repair [100].

After active or passive release from damaged or dead cells, HMGB1 is considered an alarmine or damage-associated molecular pattern molecule (DAMPs) that produces inflammation and elicits immune responses [101]. Secreted HMGB1 can be distinguished from passively released HMGB1 because it is acetylated [102]. HMGB1 binds several extracellular receptors, with the receptors for advanced glycation end products (RAGE) and Toll-like receptors (TLR) being the most studied [96]. HMGB1 activates macrophages and dendritic cells to release TNF-α and produce inflammatory cytokines and chemokines via the TLR4/MD2/MyD88/NFκB pathway [103]. HMGB proteins also activate other cell signaling pathways, including PI3K/Akt/mTOR [104].

Human HMGB1 has been investigated in many chronic disorders and the number of publications about their role in cancer has reached higher than 1000 in the last years [96]. Aberrant release of HMGB1 has been shown in human cancers [104], and HMGB1 mediates the epithelial to mesenchymal transition (EMT), which is necessary for invasion and migration in cancers from epithelial origin [84]. Besides, HMGB1 expression has been positively correlated to cisplatin resistance [105].

HMGB1 is considered a double-edged sword in cancer development since pro- and anti-oncogenic effects have been reported [106]. Through its binding to RAGE and TLR receptors, it can enhance inflammatory responses, which, if they become chronic, favor oncogenesis [104]. During hypoxia, HMGB1 up-regulates mitochondrial biogenesis in human hepatocellular carcinoma, promoting tumor survival and proliferation [107]. Hypoxia also increases HMGB1 release and RAGE expression in the tumor microenvironment, inducing the expression of proangiogenic growth factors, such as vascular endothelial growth factor (VEGF), and their receptors [106]. Anti-tumor effects of HMGB1 are produced through its interaction with tumor suppressor factors or increasing genome stability and autophagy [108,109].

HMGB1 not only activates responses to tissue damage via inflammation but also participates in tissue repair [102]—for instance, in muscle regeneration after injury [110]. Indeed, HMGB1 is considered a cytokine underscoring multiple roles in the complex response to cell damage [102]. HMGB1 stimulates innate and adaptive immunity [102,111,112] and has a dual role in relation to immune responses. HMGB1 has immunosuppressive and immune stimulatory activities, depending on redox state, receptors, and targeted cells [113]. Some anti-cancer therapies cause immunogenic cell death (ICD), which increases the immunogenicity of the cancer cells and, therefore, unleashes an adaptive immune response against the tumor and allows immunological memory [114]. It has been proposed that HMGB1 secreted by cells undergoing ICD activates dendritic cells to cross-present tumor neoantigens to lymphocytes, which elicit B- and T-cell responses [102]. HMGB1 induces apoptosis in monocyte-lineage immune cells and inhibits tumor-infiltrating macrophages and dendritic cells, lymph node sinus macrophages and liver Kupffer cells to attenuate anti-cancer immune responses, and anti-metastatic organ defense [115]. Moreover, HMGB1 fosters hepatocellular carcinoma immune evasion by promoting regulatory B-cell expansion [116]. HMGB1 is also related with the programmed cell death-1 (PD-1) receptor and its ligand (PD-L1), which negatively regulate immune cell activation [117]. PD-L1 is frequently expressed in many tumors to suppress anti-tumor immunity mediated by PD-1 positive tumor-infiltrating cytotoxic T lymphocytes through PD-L1/PD-1 ligation [118]. Nano-DOX (a delivery form of doxorubicin) stimulates the tumor cells and the tumor-associated macrophages (TAMs) to release the cytokine HMGB1, which, through the RAGE/NF-κB pathway, induce PD-L1 in the tumor cells and PD-L1/PD-1 in the tumor-associated macrophages [117]. Blockade of Nano-DOX-induced PD-L1, both in the cancer cells and the TAMs by BMS-1, achieves enhanced activation of TAM-mediated anti-tumor response [117].

From all the above, it can be deduced that HMGB proteins participate directly or indirectly in many of the hallmarks of cancer and play a significant role in the design of new therapies.

## 6. Studying HMGB Proteins: From Yeasts to Cancer

**Figure 4:**
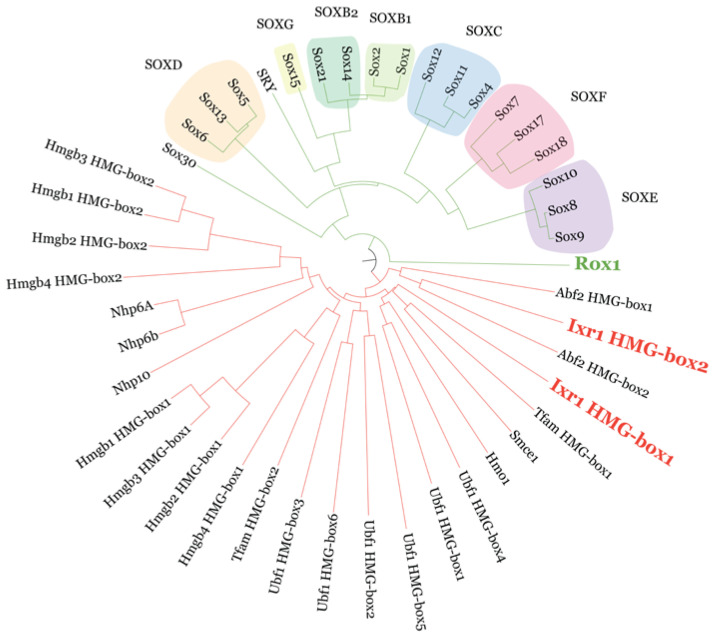
Phylogenetic relations between the HMG-box domains of the yeast proteins Rox1 and Ixr1, and other human HMGB proteins. Phylogeny analysis was made following the Neighbor-joining method (excluding gaps). Output results are available in the Supplementary File S2.

*S. cerevisiae* can grow in aerobic and anaerobic conditions, and when oxygen levels decrease, a series of genes are activated that allow yeast to adapt better to those conditions [119]. Among transcriptional regulators of hypoxic genes, Rox1 has the particularity that it is an aerobically expressed repressor that recognizes specific regulatory sequences in the promoters of hypoxic genes [119,120,121]. Structurally, Rox1 is a protein that binds DNA through its unique HMG-box [122]. From an evolutionary point of view, the HMG-box present in Rox1 from *S. cerevisiae* is related to the HMG-box present in the family of SOX transcriptional factors (Figure 4) of higher eukaryotes [90]. In vertebrates, the SOX genes characterized so far regulate developmental processes, organogenesis, and tissue homeostasis [123].

Another HMG-box protein from *S. cerevisiae*, Ixr1 (encoded by the *IXR1* gene, alias *ORD1*), controls the expression of hypoxic genes in *S. cerevisiae* by a different pathway to the one reported for Rox1 [124,125]. Ixr1 contains two HMG-boxes, which are evolutionary related to those present in HMGB proteins (Figure 4) from higher eukaryotes [90]. We found that there is a cross-regulation between the genes encoding the two HMG-box proteins Ixr1 and Rox1 in *S. cerevisiae* [126]. During aerobic growth, Ixr1 functions as a repressor of hypoxic genes, but during hypoxia, Ixr1 expression increases and preferentially acts as an activator of target genes [126,127]. We demonstrated that the NH_2_-terminal region of Ixr1 is involved in transcriptional activation and that Ixr1 binds to Ssn8 (*alias* Srb11) [128]. Ssn8 is a cyclin that interacts with Ssn3 kinase (*alias* Srb10). The Srb10-Srb11 complex contributes to transcriptional repression of diversely regulated genes in *S. cerevisiae* [129], while the Srb8-Srb9-Srb10-Srb11 complex, associated with the Mediator coactivator, functions with the SAGA complex during Gal4-activated transcription [129].

Curiously, Ixr1 has a dual life, and Lippard´s laboratory has seen that Ixr1 binds to platinated DNA and confers yeast resistance to cisplatin, with this compound and other Pt-derivatives being of clinical relevance since they are used in cancer chemotherapy [130]. It was postulated that Ixr1 does not bind specific DNA sequences but recognizes superstructures in the DNA adducts with cisplatin [131,132]. Thus, Ixr1 can recognize specific sequences in the promoters of its target genes, acting as a transcriptional regulator, but it can also behave as a protein binding DNA by other characteristics unrelated to recognition of a specific DNA sequence. A detailed study of the binding characteristics of the two HMG-boxes of Ixr1 allowed us to find a mechanism explaining how the two HMG-boxes present in the protein combine their specific characteristics to fulfill both functions [133]. 

We also studied these two HMGB proteins (Rox1 and Ixr1) in *Kluyveromyces lactis*, a non-conventional yeast classified as a respiratory yeast. Contrary to *S. cerevisiae*, *K. lactis* is unable to grow under strictly anaerobic conditions [134,135], although it can ferment sugars in hypoxic conditions with low energy efficiency [16,17]. If the sequence of these proteins is compared in *S. cerevisiae* and *K. lactis*, conservation is restricted to HMG-boxes. *Kl*Rox1 from *K. lactis* does not regulate the hypoxic response in this yeast but it is involved in the oxidative stress response produced by arsenate and cadmium [136]. The *Sc*Ixr1 and *Kl*Ixr1 proteins have several conserved functions in the control of gene expression; however, we found major differences between *Sc*Irx1 and *Kl*Ixr1 affecting cellular responses to cisplatin [137]. 

Further studies carried out to analyze the regulatory effects of *IXR1* gene deletion upon gene transcription in *S. cerevisiae* showed that Ixr1 is a master regulator that controls the expression of other transcriptional factors that respond to nutrient availability or stress stimuli and are related to the TOR pathway and PKA signaling [138]. Ribosome biogenesis in *S. cerevisiae* involves a regulon of >200 genes (Ribi genes) coordinately regulated in response to nutrient availability and cellular growth rate. As confirmed by chromatin immunoprecipitation (ChIP) and expression analyses, Ixr1 controls transcription of ribosomal RNAs and genes encoding ribosomal proteins (RBPs) or that are involved in ribosome assembly. In summary, Ixr1 controls gene expression involved in ribosome biogenesis by direct binding to target promoters, or by indirect mechanisms, modulating the expression of other transcriptional factors. Cisplatin treatment mimics the effect of IXR1 deletion on rRNA and RBPs gene transcription, and prevents Ixr1 binding to specific promoters related to these processes, kidnapping the Ixr1 protein to cisplatin-DNA adducts with higher affinity than promoter regulatory sequences [133,138]. Ribosome biogenesis needs the coordinated and balanced production of mRNAs, rRNAs, and Ribi-proteins, and distortion of this balance generates ribosome biogenesis alterations that can impact cell cycle progression (reviewed by [139]). Sato and collaborators also found that Ixr1 is directly involved in cell cycle progression; *IXR1* mRNA is a physiologically important target of Puf5, and cell cycle progression in *S. cerevisiae* is modulated by these factors through the regulation of the cell-cycle-specific expression of *CLB1* [140].

Taking a huge leap in evolution, and moving from the humble yeast to the complex human system, we can find certain functional parallels between yeast Ixr1 and human p53. The p53 protein is coded by the TP53 gene, which is the most frequently mutated gene in human tumors [141]. Both proteins are transcriptional factors whose levels, stability, or activity are increased during hypoxia: Ixr1 by a cross talk with Rox1 [126], and p53 by direct and indirect interactions with Hypoxia Inducible Factor-1 (HIF-1) [142]. Both respond to genotoxic stress and are involved in DNA repair [143]. Both are related to ribosome biogenesis and cell cycle control [138,140,144,145]. Stabilization of p53 upon DNA damage is followed by reversible or irreversible cell cycle arrest or programmed cell death; p53 also responds to non-genotoxic cell stress if ribosome biogenesis is affected [146], and several ribosomal proteins can activate the p53 tumor suppressor pathway [144]. However, p53 is not structurally related to Ixr1 and is not a HMGB protein, therefore we looked for other human proteins with the structural HMG-box domain and that might interact with p53. The laboratory of Jean O. Thomas published that HMGB1 interacts with the N-terminal region of p53 through its HMGB-box domain and facilitates the binding of p53 to DNA by its HMG-boxA [147]. HMGB1 over-expression is extensively associated with cancer, including those of the prostate and ovary [94,148], and it has been demonstrated that HMGB1 silencing slows cell growth and inhibits the growth of xenograft tumors in nude mice [149].

Taking advantage of our expertise using yeast tools, we carried out a Y2H approach to characterize proteins interacting with human HMGB1 and HMBG2 in prostate cancer [35] and ovarian cancer [91] cells; in both studies, we have found connections to ribosome biogenesis control. In the study of ovarian cancer, we have characterized the interaction of HMGB2 with Nop53 [91], a ribosome assembly factor that has a structural role in the formation of nuclear pre-60S intermediates, affecting late maturation events [150]. Nop53 translocates to the nucleoplasm under ribosomal stress, where it interacts and stabilizes p53 and inhibits cell cycle progression [150]. In the study of prostate cancer, we also found that HMGB2 interacts with Nop53 and with Rps28; the latter is related to the assembly of 40S ribosomal subunits [151].

To extend the number of targets detected in the Y2H interactomes, we also carried out a HMGB1-interactome analysis approach based on immunoprecipitation (IP) and mass spectrometry (MS) in prostate and ovary cancer cell lines. The corresponding HMGB1 nuclear interactomes were clearly enriched in mRNA and rRNA processing factors [152]. The interaction of HMGB1 with the subunit Rbbp7 of the Nucleosome Remodeling (NuRD) complex was validated and other subunits of this complex were also identified in the IPs, including the histone deacetylases HDAC1 and HDAC2 [152]. The Upstream binding factor (UBF) is responsible for the recruitment of the RNA PolI pre-initiation complex required for rRNA transcription. It has been reported that deacetylation of UBF by HDAC1 disrupts the recruitment of UBF to PolI and causes a decrease in rDNA transcription, thus affecting cell proliferation [153]. In the prostate cancer cell line PC-3, silencing of the HMGB1 gene induced downregulation of key regulators of ribosome biogenesis and RNA processing such as OP1, RSS1, UBF1, KRR1, and LYAR. The analysis carried out using results from databases revealed that upregulation of these genes in prostate adenocarcinomas correlates with worse prognosis, reinforcing their functional significance in cancer progression [152].

## 7. Ongoing Yeast Perspectives in Biomedicine

The knowledge acquired finding interactions of HMGB proteins with targets that control ribosome biogenesis, cell cycle, and proliferation of cancerous cells has led us to continue new projects to detect these markers in liquid biopsies as well as to find other molecules such as lncRNAs that interact with HMGB proteins and enhance or inhibit these processes. Several lncRNAs are deregulated in cancer [154], and RIP (RNA immunoprecipitation) and eCLIP (enhanced crosslinking and immunoprecipitation) assays have been carried out in ovary cancer cells to confirm putative interactions with HMGB proteins (unpublished data from our laboratory). We are also using yeast cell surface display screening to find neoantigens stimulating CRC-receptors specific of B-lymphocytes infiltrating in ovary tumors to potentiate the immune response against malignant cells.

Yeasts are also being used nowadays in the field of medicine to produce nanobodies, which are monomeric antigen-binding domains derived from the camelid heavy chain-only antibodies [155] and affibodies—small imitating monoclonal antibodies that bind with high affinity [156]. Yeasts models are also being used in aging research [157], and humanized yeasts allow the measurement of human protein activity in a cheaper and simplified model [158]. The potential of Single Molecule Tracking (SMT) in yeast, a method of choice for the biochemical characterization of protein dynamics in vitro and in vivo, has been recently evidenced [159].

The long history of yeasts in science, their valuable contributions to research, and the broad perspectives of their use in new fields make us think that they will continue to accompany scientists for many years, contributing to the improvement of human life, as they have from the beginning. 

## Data Availability

Not applicable.

## Supplementary Materials

The following supporting information can be downloaded at: https://www.mdpi.com/article/10.3390/microorganisms11040993/s1, S1: Alignment of sequences used to build up Figure 2; S2: Phylogeny analysis of HMG-boxes by the Neighbour-joining method (excluding gaps): Out-put results.
