# Supplementary material for: Thanksgiving to Yeast, the HMGB Proteins History from Yeast to Cancer
Source: Microorganisms. 2023 Apr 11;11(4):993. doi: 10.3390/microorganisms11040993 (PMC10142021; doi:10.3390/microorganisms11040993)
Supplement: Supplementary file 1 [file microorganisms-11-00993-s001.zip › microorganisms-2283444-supplementary.pdf]

Supplementary S1.- Alignment of sequences used to build up Figure 2

>Ubf1HMG-box3
TKPPPNSYSLYCAELMAN--MKDVPST-------
ERMVLCSQQWKLLSQKEKDAYHKKCDQKKKDYEVELLRFL
>Ubf1HMG-box6
KKPPMNGYQKFSQELLSNGELNHLPLK--------
ERMVEIGSRWQRISQSQKEHYKKLAEEQQKQYKVHLDLWV
>Ubf1HMG-box2
PEKPKTPQQLWYTHEKKV-YLKVRPDA-----
TTKEVKDSLGKQWSQLSDKKRLKWIHKALEQRKEYEEIMRDYI
>Ubf1HMG-box5
PESPKRAEEIWQQSVIGD-YLARFKND-----RV-
KALKAMEMTWNNMEKKEKLMWIKKAAEDQKRYERELSEMR
>Hmo1
PKKPLTVFFAYSAYVRQE-
LREDRQKAGLPPLSSTEITQEISKKWKELSDNEKEKWKQAYNVELENYQREKSKYL
>Ubf1HMG-box1
PKKPLTPYFRFFMEKRAK-YAKLHPEM-----
SNLDLTKILSKKYKELPEKKKMKYIQDFQREKQEFERNLARFR
>Ixr1HMG-box1
PKRPSSAYFLFSMSIRNE-LLQQFPEA-----
KVPELSKLASARWKELTDDQKKPFYEEFRTNWEKYRVVRDAYE
>Rox1
IPRPKNAFILFRQHYHRI-
LIDEWTAQGVEIPHNSNISKIIGTKWKGLQPEDKAHWENLAEKEKLEHERKYPEYK
>Sox30
VKRPMNAFMVWARIHRPA-LAKANPAA-----
NNAEISVQLGLEWNKLSEEQKKPYYDEAQKIKEKHREEFPGWV
>Sox5
IKRPMNAFMVWAKDERRK-ILQAFPDM-----
HNSNISKILGSRWKAMTNLEKQPYYEEQARLSKQHLEKYPDYK
>Sox13
IKRPMNAFMVWAKDERRK-ILQAFPDM-----
HNSSISKILGSRWKSMTNQEKQPYYEEQARLSRQHLEKYPDYK
>Sox6
IKRPMNAFMVWAKDERRK-ILQAFPDM-----
HNSNISKILGSRWKSMSNQEKQPYYEEQARLSKIHLEKYPNYK
>SRY
VKRPMNAFIVWSRDQRRK-MALENPRM-----
RNSEISKQLGYQWKMLTEAEKWPFFQEAQKLQAMHREKYPNYK
>Sox15
VKRPMNAFMVWSSAQRRQ-MAQQNPKM-----
HNSEISKRLGAQWKLLDEDEKRPFVEEAKRLRARHLRDYPDYK
>Sox14
IKRPMNAFMVWSRGQRRK-MAQENPKM-----
HNSEISKRLGAEWKLLSEAEKRPYIDEAKRLRAQHMKEHPDYK
>Sox21
VKRPMNAFMVWSRAQRRK-MAQENPKM-----
HNSEISKRLGAEWKLLTESEKRPFIDEAKRLRAMHMKEHPDYK
>Sox1
VKRPMNAFMVWSRGQRRK-MAQENPKM-----
HNSEISKRLGAEWKVMSEAEKRPFIDEAKRLRALHMKEHPDYK
>Sox2
VKRPMNAFMVWSRGQRRK-MAQENPKM-----
HNSEISKRLGAEWKLLSETEKRPFIDEAKRLRALHMKEHPDYK
>Sox12

IKRPMNAFMVWSQHERRK-IMDQWPDM-----
HNAEISKRLGRRWQLLQDSEKIPFVREAERLRLKHMADYPDYK
>Sox4
IKRPMNAFMVWSQIERRK-IMEQSPDM-----
HNAEISKRLGKRWKLLKDSDKIPFIREAERLRLKHMADYPDYK
>Sox11
IKRPMNAFMVWSKIERRK-IMEQSPDM-----
HNAEISKRLGKRWKMLKDSEKIPFIREAERLRLKHMADYPDYK
>Sox10
VKRPMNAFMVWAQAARRK-LADQYPHL-----
HNAELSKTLGKLWRLLNESDKRPFIEEAERLRMQHKKDHPDYK
>Sox9
VKRPMNAFMVWAQAARRK-LADQYPHL-----
HNAELSKTLGKLWRLLNESEKRPFVEEAERLRVQHKKDHPDYK
>Sox8
VKRPMNAFMVWAQAARRK-LADQYPHL-----
HNAELSKTLGKLWRLLSESEKRPFVEEAERLRVQHKKDHPDYK
>Sox7
IRRPMNAFMVWAKDERKR-LAVQNPDL-----
HNAELSKMLGKSWKALTLSQKRPYVDEAERLRLQHMQDYPNYK
>Sox18
IRRPMNAFMVWAKDERKR-LAQQNPDL-----
HNAVLSKMLGKAWKELNAAEKRPFVEEAERLRVQHLRDHPNYK
>Sox17
IRRPMNAFMVWAKDERKR-LAQQNPDL-----
HNAELSKMLGKSWKALTLAEKRPFVEEAERLRVQHMQDHPNYK
>Nhp10
PKRPTNAYLLYCEMNKER--IRQNGSL--------
DVTRDLAEGWKNLNEQDRKPYYKLYSEDRERYQMEMEIYN
>Hmgb4HMG-box1
PKANVSSYVHFLLNYRNK-FKEQQPNT---
YVGFKEFSRKCSEKWRSISKHEKAKYEALAKLDKARYQEEMMNYV
>Hmgb2HMG-box1
PKGKMSAYAFFVQTCREE-HKKKNPEV---
PVNFAEFSKKCSERWKTMSGKEKSKFDEMAKADKVRYDREMKDYG
>Hmgb1HMG-box1
PRGKMSSYAFFVQTCREE-HKKKHPDA---
SVNFSEFSKKCSERWKTMSAKEKGKFEDMAKADKARYEREMKTYI
>Hmgb3HMG-box1
PRGKMSSYAFFVQTCREE-HKKKHPDS---
SVNFAEFSKKCSERWKTMSAKEKSKFEDMAKSDKARYDREMKNYV
>Ubf1HMG-box4
PKRPVSAMFIFSEEKRRQ-LQEERPEL-----
SESELTRLLARMWNDLSEKKKAKYKAREAALKAQSERKPGGER
>Nhp6A
PKRALSAYMFFANENRDI-VRSENPDI-----
TFGQVGKKLGEKWKALTPEEKQPYEAKAQADKKRYESEKELYN
>Nhp6b
PKRGLSAYMFFANENRDI-VRSENPDV-----
TFGQVGRILGERWKALTAEEKQPYESKAQADKKRYESEKELYN
>Hmgb4HMG-box2
PRRPPSSFLLFCQDHYAQ-LKRENPNW-----
SVVQVAKATGKMWSTATDLEKHPYEQRVALLRAKYFEELELYR
>Hmgb2HMG-box2
PKRPPSGFFLFCSEFRPK-IKSTNPGI-----
SIGDVAKKLGEMWNNLNDSEKQPYITKAAKLKEKYEKDVADYK
>Hmgb3HMG-box2

```
PKRPPSAFFLFCSEHRPK-IKSEHPGL-----
SIGDTAKKLGEMWSEQSAKDKQPYEQKAAKLKEKYEKDIAAYR
>Hmgb1HMG-box2
PKRPPSAFFLFCSEYRPK-IKGEHPGL-----
SIGDVAKKLGEMWNNTAADDKQPYEKKAAKLKEKYEKDIAAYR
>Smce1
PDKPLMPYMRYSRKVWDQ-VKASNPDL-----
KLWEIGKIIGGMWRDLTDEEKQEYLNEYEAEKIEYNESMKAYH
>TfamHMG-box1
PKKPVSSYLRFSKEQLPI-FKAQNPDA-----
KTTELIRRIAQRWRELPDSKKKIYQDAYRAEWQVYKEEISRFK
>TfamHMG-box2
PKRPRSAYNVYVAERFQE-AKGDSPQ---------
EKLKTVKENWKNLSDSEKELYIQHAKEDETRYHNEMKSWE
>Abf2HMG-box1
PKRPTSAYFLYLQDHRSQ-FVKENPTL-----
RPAEISKIAGEKWQNLEADIKEKYISERKKLYSEYQKAKKEFD
>Abf2HMG-box2
PKKPAGPFIKYANEVRSQ-VFAQHPDK-----
SQLDLMKIIGDKWQSLDQSIKDKYIQEYKKAIQEYNARYPLN-
>Ixr1HMG-box2
PKRPSGPFIQFTQEIRPT-VVKENPDK-----
GLIEITKIIGERWRELDPAKKAEYTETYKKRLKEWESCYPDEN
```

Supplementary S2.-Phylogeny analysis of HMG-boxes by the Neighbour-joining method (excluding gaps): Out-put results

```
(
(
(
(
(
Ubf1HMG-box3:0.31429,
Ubf1HMG-box6:0.33186)
:0.06548,
(
Ubf1HMG-box2:0.35106,
Ubf1HMG-box5:0.38423)
:0.04910)
:0.01635,
(
(
Nhp10:0.36012,
(
(
Nhp6A:0.04762,
Nhp6b:0.05383)
:0.24876,
(
Hmgb4HMG-box2:0.30762,
(
Hmgb2HMG-box2:0.11615,
(
Hmgb3HMG-box2:0.06252,
Hmgb1HMG-box2:0.06791)
:0.06501)
:0.14166)
:0.04109)
:0.01205)
:0.02018,
(
(
Hmgb4HMG-box1:0.27572,
(
Hmgb2HMG-box1:0.08951,
(
Hmgb1HMG-box1:0.04726,
Hmgb3HMG-box1:0.05133)
:0.05837)
:0.16794)
:0.07249,
TfamHMG-box2:0.31792)
:0.02652)
:0.00714)
:0.01112,
(
(
Rox1:0.35254,
(
Sox30:0.28078,
(
```

```
(
(
Sox5:0.02749,
Sox13:0.03048)
:0.00543,
Sox6:0.03805)
:0.19598,
(
(
SRY:0.17418,
(
Sox15:0.10556,
(
(
Sox14:0.02533,
(
Sox1:0.02739,
Sox2:0.01609)
:0.01815)
:0.00806,
Sox21:0.03904)
:0.06111)
:0.05771)
:0.04252,
(
(
Sox12:0.06447,
(
Sox4:0.02318,
Sox11:0.02030)
:0.03698)
:0.11600,
(
(
Sox10:0.02869,
(
Sox9:0.00903,
Sox8:0.00546)
:0.01479)
:0.13891,
(
Sox7:0.07344,
(
Sox18:0.05575,
Sox17:0.04570)
:0.02801)
:0.09388)
:0.02395)
:0.03482)
:0.00573)
:0.05736)
:0.03967)
:0.05347,
(
Abf2HMG-box1:0.30367,
(
Abf2HMG-box2:0.29943,
Ixr1HMG-box2:0.28881)
```

```
:0.04448)
:0.02431)
:0.01678)
:0.01063,
(
Hmo1:0.35338,
(
Ubf1HMG-box1:0.32325,
Ubf1HMG-box4:0.31443)
:0.03793)
:0.01375,
(
Ixr1HMG-box1:0.34981,
(
Smce1:0.31777,
TfamHMG-box1:0.31991)
:0.04150)
:0.00256);
```
